# Supplementary material for: A tale of two vineyards: parsing site-specific differences in bacterial and fungal communities of wine grapes from proximal vineyards and their changes during processing in a single winery
Source: Appl Environ Microbiol. 2025 May 5;91(6):e00526-25. doi: 10.1128/aem.00526-25 (PMC12175509; doi:10.1128/aem.00526-25)
Supplement: Supplemental material — Figures S1 to S14; Tables S1 and S2. [file aem.00526-25-s0001.pdf]

Supplemental Material for: Differences Between Fruit-Associated Microbial Communities from Proximal Vineyards, and Their Changes Through Processing.

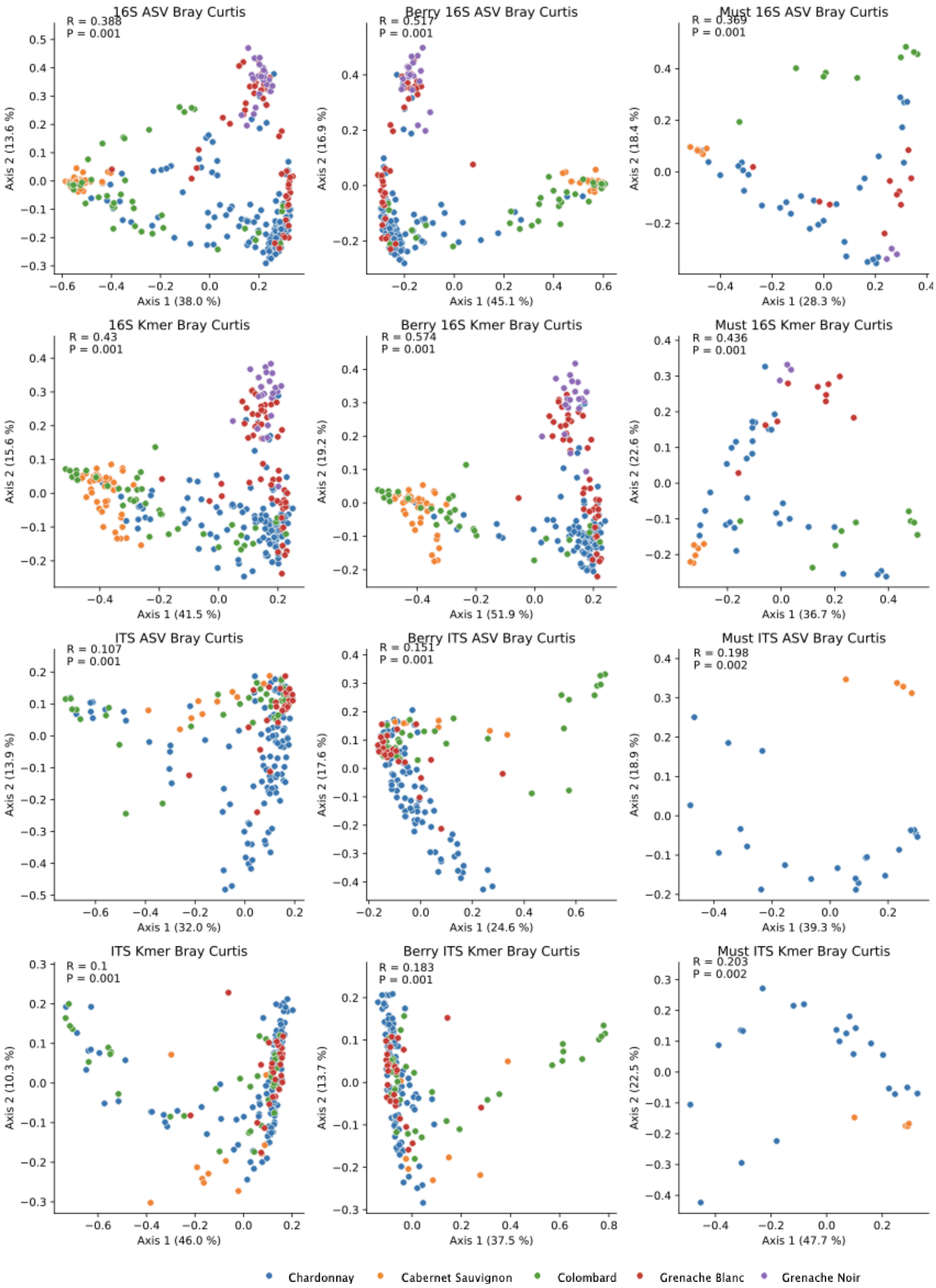

Figure S1. Grape varieties retain distinct microbiota composition through processing. Bray-Curtis dissimilarity PCoA Plots of grape and must (left), grape berry (middle) and must samples (right) based on bacterial (16S) and fungal (ITS) composition; and colored by variety. Blue = Chardonnay; Orange = Cabernet Sauvignon; Green = Colombard; Red = Grenache Blanc; Purple = Grenache Noir. Inset: PERMANOVA R- and P-values.

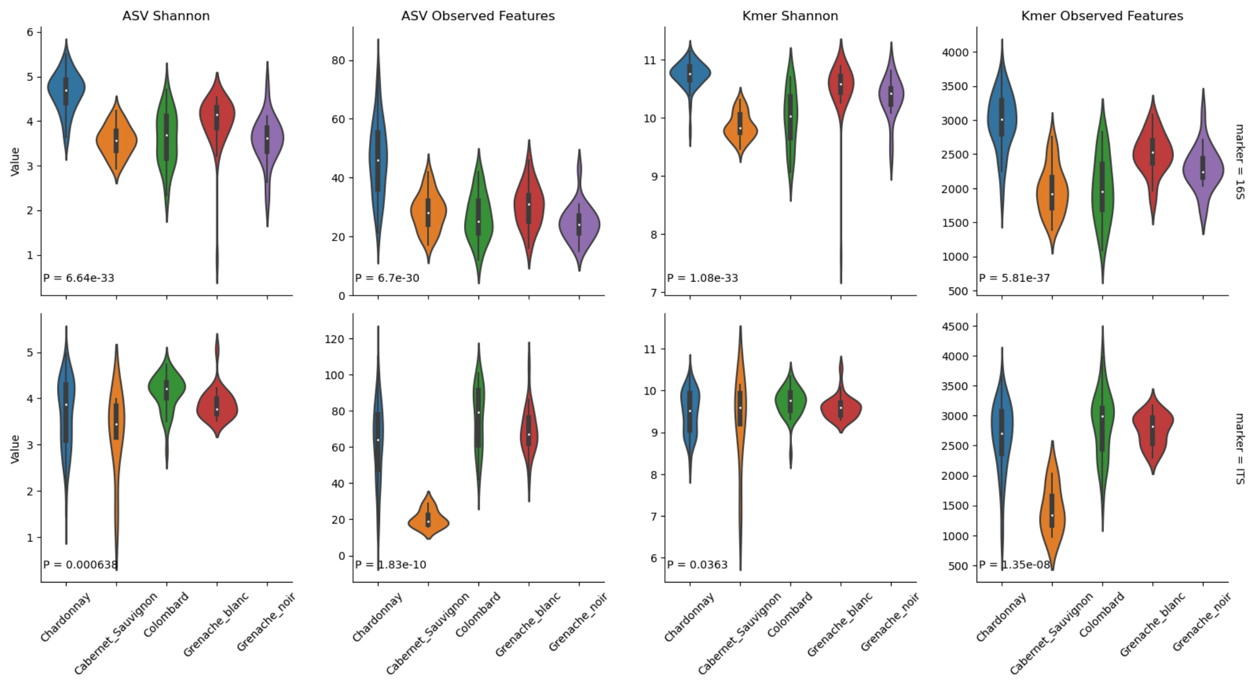

Figure S2. Alpha diversity varies significantly between grapevine varieties. Shannon H and observed feature counts (richness) of ASVs (left two columns) and k-mers (right two columns) are shown for bacterial communities (top row) and fungal communities (bottom row) for five grapevine varieties from Tyree and RMI vineyards. P-values indicate the result of one-way ANOVA tests comparing all varieties.

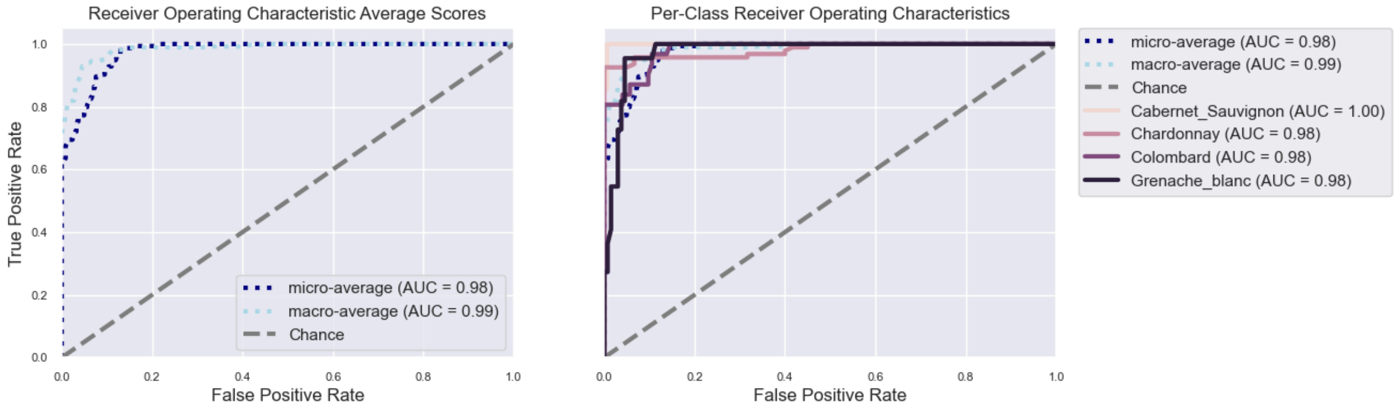

Figure S3. Random Forest classifier performance for predicting grapevine variety from bacterial and fungal community composition. Receiver-operating characteristic (ROC) curves show model accuracy as the relationship between the true positive rate (TPR, y-axis) and the false positive rate (FPR, x-axis) at various threshold settings. Area under the curve (AUC) indicates model performance, with AUC=1.0 being perfect accuracy. The grey dashed line indicates the performance expected from random guessing. Note: Grenache Noir is not present in this analysis as these samples failed ITS sequencing and could not be included in the merged tables. Supervised classification was performed on merged bacterial and fungal ASVs. Only samples represented in both were included in this analysis.

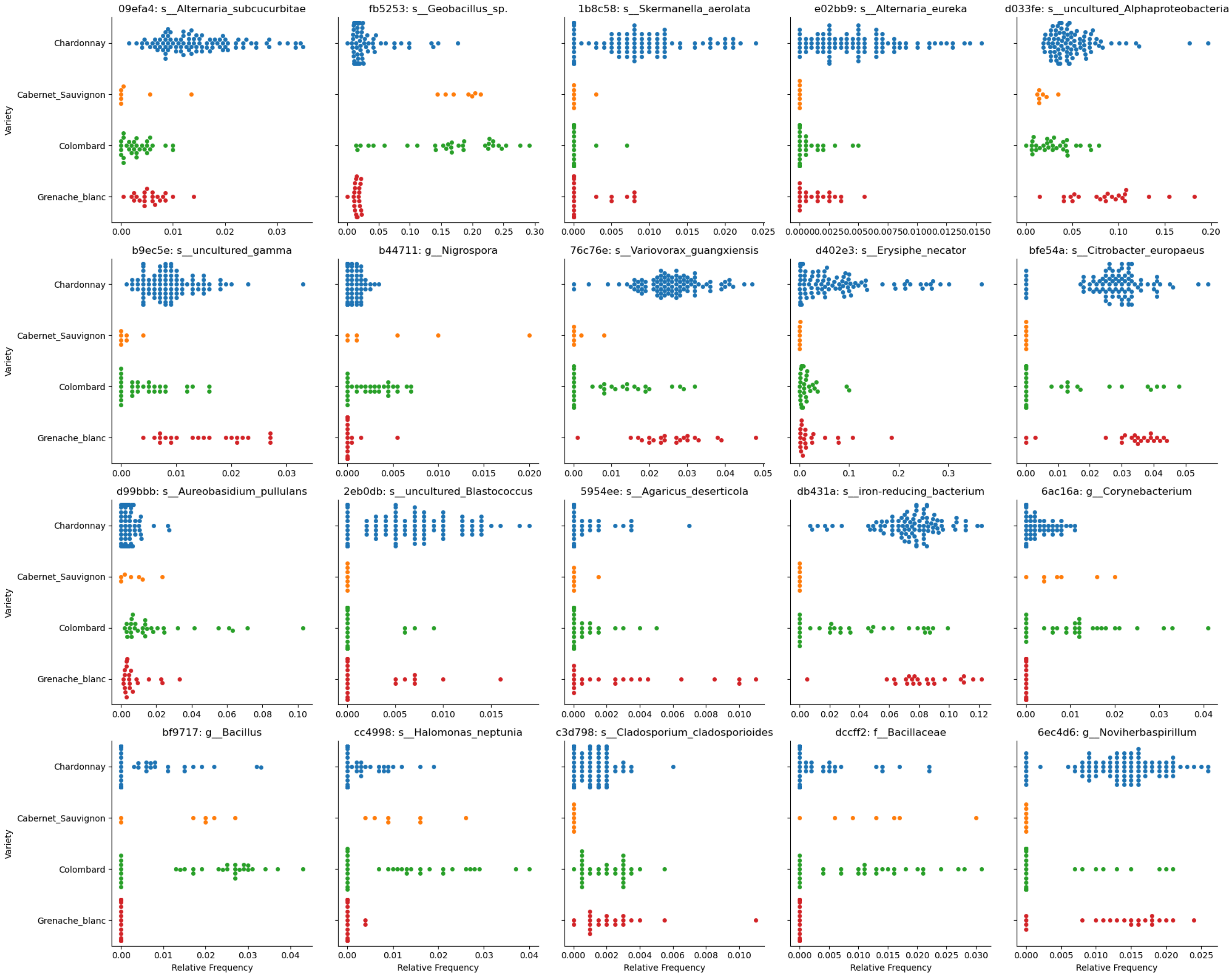

Figure S4. Relative frequency of the top 20 predictive features (ASVs) identified by Random Forest classification for differentiating grapevine varieties. Stripplots show the relative abundance of each taxon in each sample (point), grouped by variety. Subpanel titles indicate the unique ASV ID (first 6 characters) followed by the species identity (or deepest taxonomic rank at which that ASV could be identified). Note: Grenache Noir is not present in this analysis as these samples failed ITS sequencing and could not be included in the merged tables. Supervised classification was performed on merged bacterial and fungal ASVs. Only samples represented in both were included in this analysis.

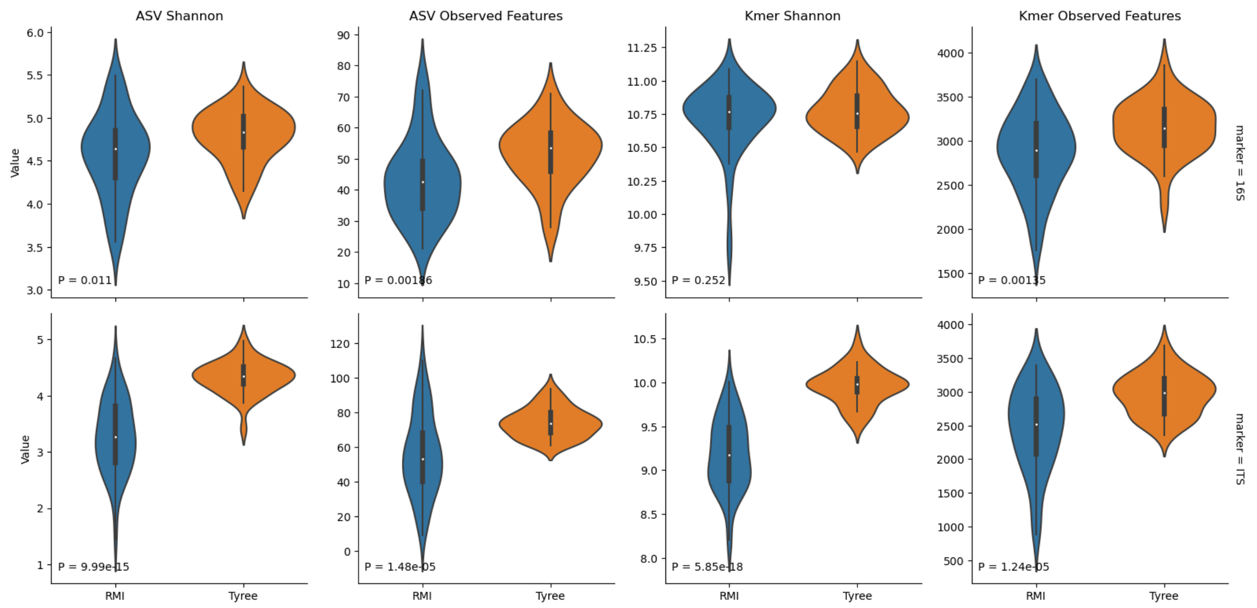

Figure S5. Alpha diversity varies significantly between Tyree and RMI vineyards. Shannon H and observed feature counts (richness) of ASVs (left two columns) and k-mers (right two columns) are shown for bacterial communities (top row) and fungal communities (bottom row) for five grapevine varieties from Tyree and RMI vineyards. P-values indicate the result of one-way ANOVA tests comparing all varieties.

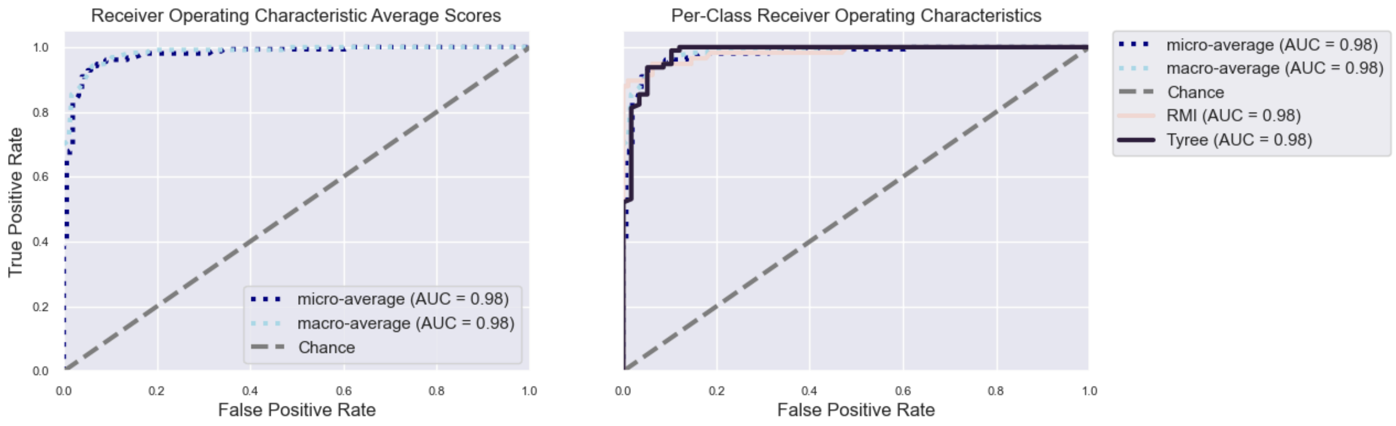

Figure S6. Random Forest classifier performance for predicting vineyard site from bacterial and fungal community composition. Receiver-operating characteristic (ROC) curves show model accuracy as the relationship between the true positive rate (TPR, y-axis) and the false positive rate (FPR, x-axis) at various threshold settings. Area under the curve (AUC) indicates model performance, with AUC=1.0 being perfect accuracy. The grey dashed line indicates the performance expected from random guessing.

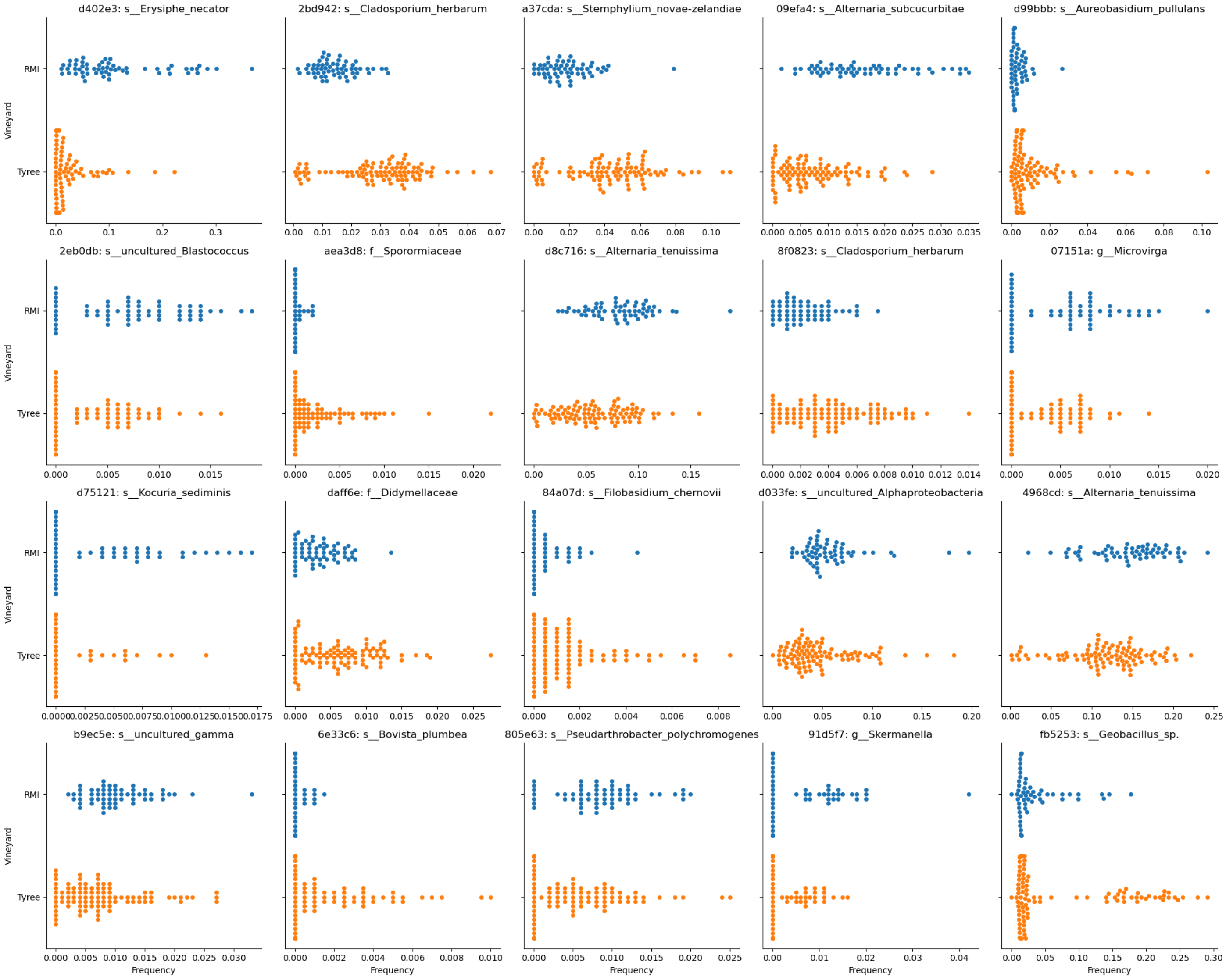

Figure S7. Relative frequency of the top 20 predictive features (ASVs) identified by Random Forest classification for differentiating vineyard sites. Stripplots show the relative abundance of each taxon in each sample (point), grouped by vineyard site. Subpanel titles indicate the unique ASV ID (first 6 characters) followed by the species identity (or deepest taxonomic rank at which that ASV could be identified).

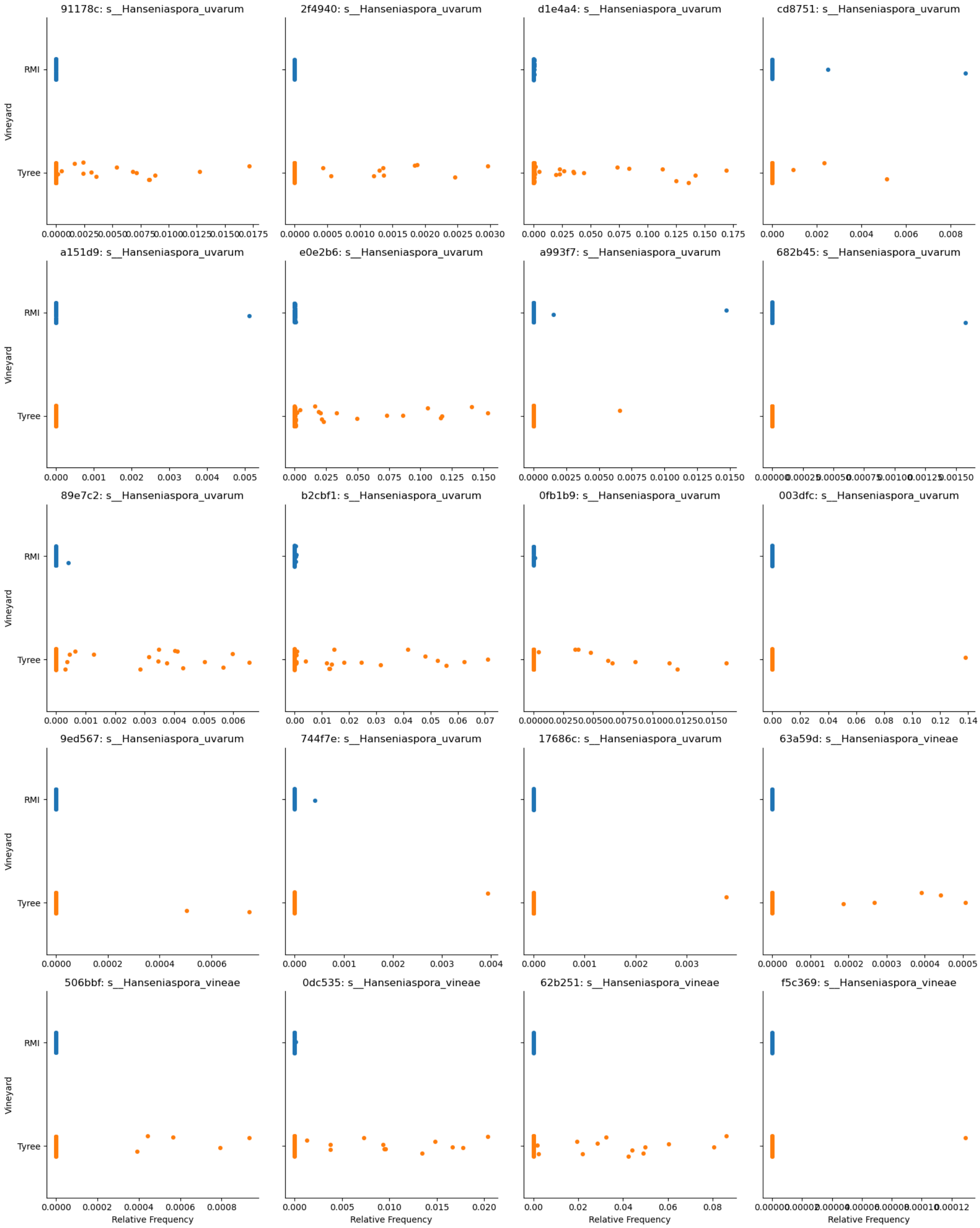

Figure S8. Relative frequency of all *Hanseniaspora* ASVs by vineyard site. Stripplots show the relative abundance of each taxon in each sample (point), grouped by vineyard site. Subpanel titles indicate the unique ASV ID (first 6 characters) followed by the species identity.

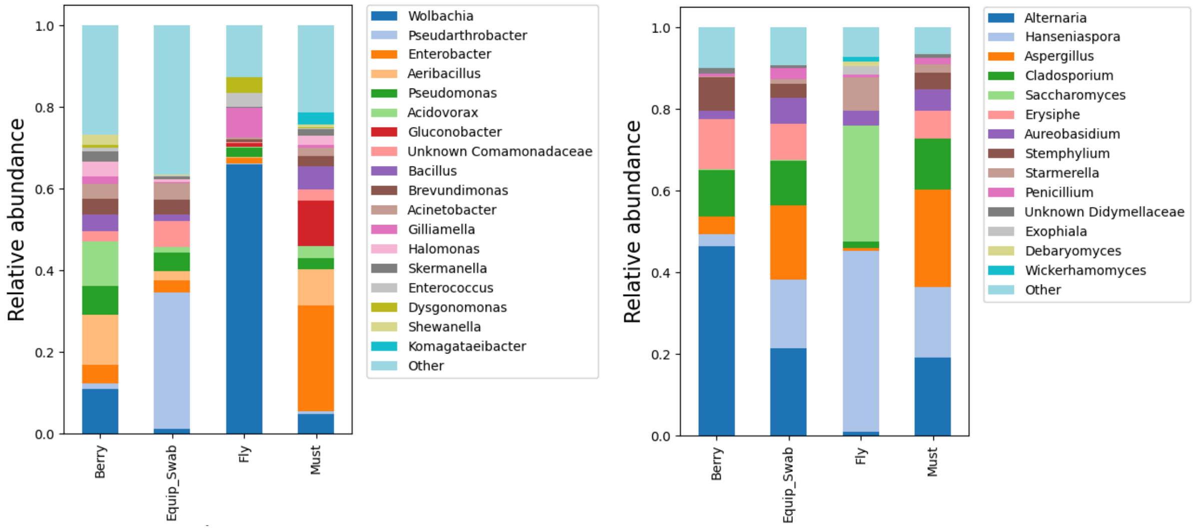

Figure S9. Mean relative abundances of bacteria (left) and fungi(right) observed in each sample type. Stacked barplots show the mean abundance of the most abundant genera, averaged by sample type. All genera observed at a mean relative abundance of < 0.025 (bacteria) or 0.01 (fungi) are binned as "Other".

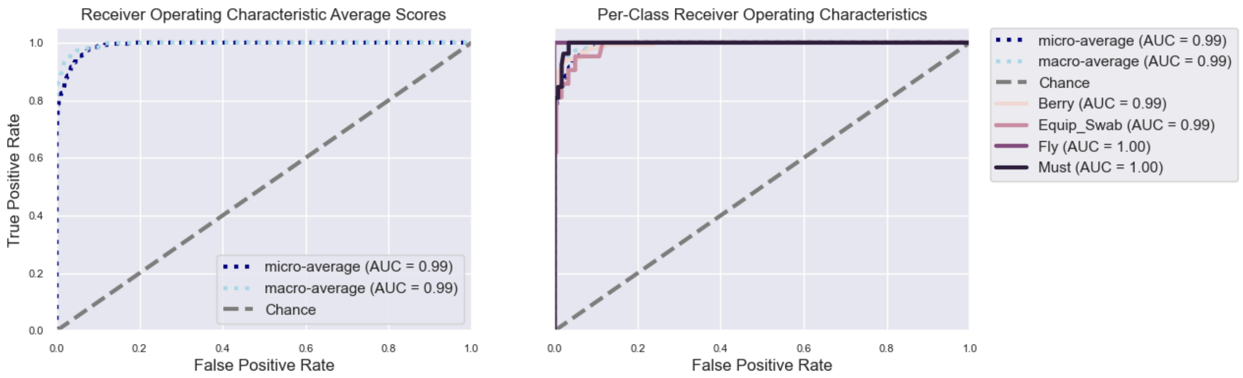

Figure S10. Random Forest classifier performance for predicting sample type from bacterial and fungal community composition. Receiver-operating characteristic (ROC) curves show model accuracy as the relationship between the true positive rate (TPR, y-axis) and the false positive rate (FPR, x-axis) at various threshold settings. Area under the curve (AUC) indicates model performance, with AUC=1.0 being perfect accuracy. The grey dashed line indicates the performance expected from random guessing.

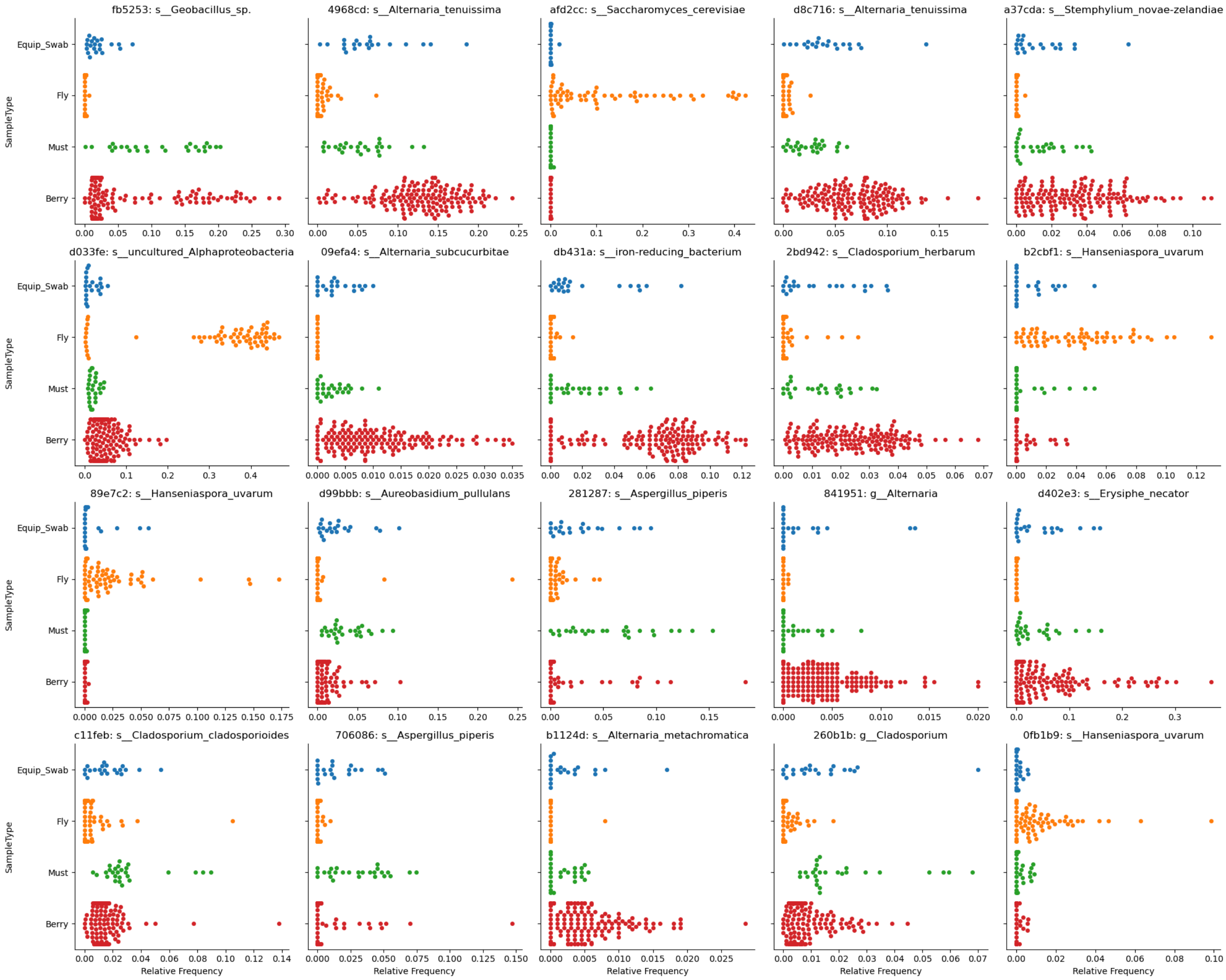

Figure S11. Relative frequency of the top 20 predictive features (ASVs) identified by Random Forest classification for differentiating sample types. Stripplots show the relative abundance of each taxon in each sample (point), grouped by sample type. Subpanel titles indicate the unique ASV ID (first 6 characters) followed by the species identity (or deepest taxonomic rank at which that ASV could be identified).

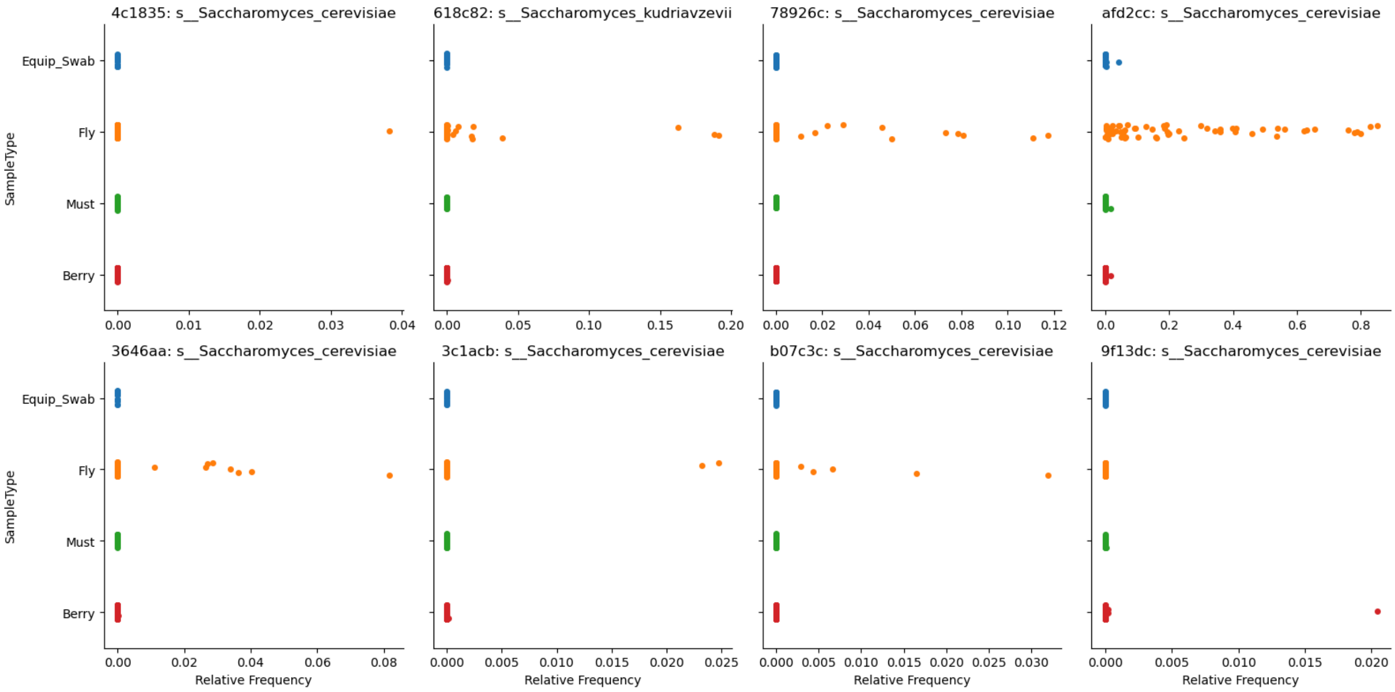

Figure S12. Relative frequency of all *Saccharomyces* ASVs by sample type. Stripplots show the relative abundance of each taxon in each sample (point), grouped by sample type. Subpanel titles indicate the unique ASV ID (first 6 characters) followed by the species identity.

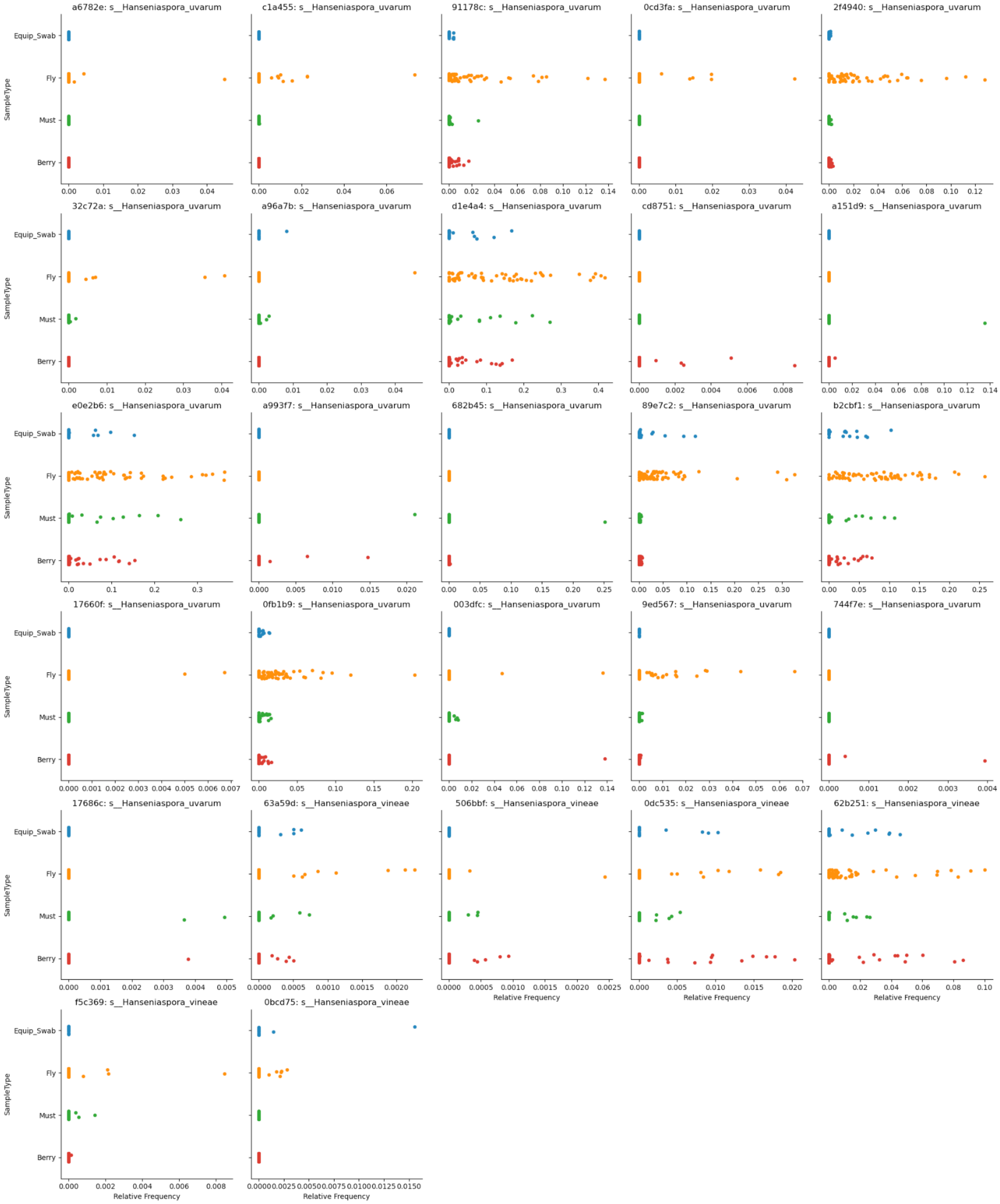
a6782e: s__Hanseniaspora_uvarum
c1a455: s__Hanseniaspora_uvarum
91178c: s__Hanseniaspora_uvarum
0cd3fa: s__Hanseniaspora_uvarum
2f4940: s__Hanseniaspora_uvarum
32c72a: s__Hanseniaspora_uvarum
a96a7b: s__Hanseniaspora_uvarum
d1e4a4: s__Hanseniaspora_uvarum
cd8751: s__Hanseniaspora_uvarum
a151d9: s__Hanseniaspora_uvarum
e0e2b6: s__Hanseniaspora_uvarum
a993f7: s__Hanseniaspora_uvarum
682b45: s__Hanseniaspora_uvarum
89e7c2: s__Hanseniaspora_uvarum
b2cbf1: s__Hanseniaspora_uvarum
17660f: s__Hanseniaspora_uvarum
0fb1b9: s__Hanseniaspora_uvarum
003dfc: s__Hanseniaspora_uvarum
9ed567: s__Hanseniaspora_uvarum
744f7e: s__Hanseniaspora_uvarum
17686c: s__Hanseniaspora_uvarum
63a59d: s__Hanseniaspora_vineae
506bbf: s__Hanseniaspora_vineae
0dc535: s__Hanseniaspora_vineae
62b251: s__Hanseniaspora_vineae
f5c369: s__Hanseniaspora_vineae
0bcd75: s__Hanseniaspora_vineae
SampleType
Equip_Swab
Fly
Must
Berry
Relative Frequency

Figure S13. Relative frequency of all *Hanseniaspora* ASVs by sample type. Stripplots show the relative abundance of each taxon in each sample (point), grouped by sample type. Subpanel titles indicate the unique ASV ID (first 6 characters) followed by the species identity.

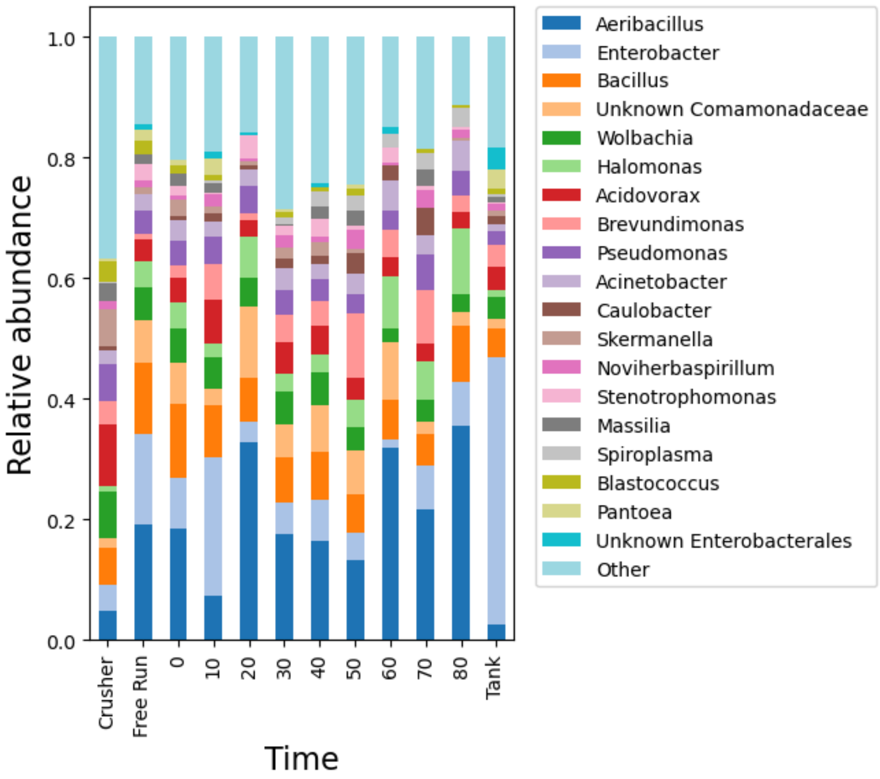

Figure S14. Dynamic changes in the bacterial community profile of grape must/juice samples during processing. Stacked barplots show the relative abundance of bacterial genera in grape must/juice samples at different press times, as well as crushed grapes and free run juice samples immediately prior to pressing, and settling tank samples immediately after. All taxa observed at < 0.03 relative abundance are binned into "Other".

Table S1. PERMANOVA tests for associating bacterial and fungal community composition of Chardonnay grape samples with vineyard source.

**Community**	**Bacteria***				**Fungi**			
**Metric**	**Jaccard**		**Bray-Curtis**		**Jaccard**		**Bray-Curtis**	
**Feature Type**	**ASV**	**K-mer**	**ASV**	**K-mer**	**ASV**	**K-mer**	**ASV**	**K-mer**
**R2 values**	0.037	0.039	0.066	0.077	0.045	0.084	0.229	0.272
**P-Values**	0.001	0.001	0.001	0.001	0.001	0.001	0.001	0.001

* Each column corresponds to a single one-way PERMANOVA test examining the association between vineyard with Chardonnay grape microbiota composition based on the corresponding metric, feature type, and amplicon target (bacterial 16S rRNA genes or Fungal ITS domain). R2 (top, corresponding to the proportion of variance explained) and P-values (bottom) are shown for each test. In this test, the input data are filtered to only contain Chardonnay grape berry samples from the two vineyards, so sample type and variety information is not included in the test formula.

Table S2. PERMANOVA tests for associating bacterial and fungal community composition of grape samples with grape variety source (Tyree vineyard only).

**Community**	**Bacteria***				**Fungi**			
**Metric**	**Jaccard**		**Bray-Curtis**		**Jaccard**		**Bray-Curtis**	
**Feature Type**	**ASV**	**K-mer**	**ASV**	**K-mer**	**ASV**	**K-mer**	**ASV**	**K-mer**
**R2 values**	0.320	0.391	0.603	0.646	0.118	0.197	0.185	0.214
**P-Values**	0.001	0.001	0.001	0.001	0.001	0.001	0.001	0.001

* Each column corresponds to a single one-way PERMANOVA test examining the association between grape variety with grape microbiota composition based on the corresponding metric, feature type, and amplicon target (bacterial 16S rRNA genes or Fungal ITS domain). R2 (top, corresponding to the proportion of variance explained) and P-values (bottom) are shown for each test. In this test, the input data are filtered to only contain only grape berry samples from Tyree vineyard, so sample type and vineyard information is not included in the test formula.
